# Examining postpartum depression screening effectiveness in well child clinics in Alberta, Canada: A study using the All Our Families cohort and administrative data

**DOI:** 10.1016/j.pmedr.2019.100888

**Published:** 2019-05-03

**Authors:** Shainur Premji, Sheila W. McDonald, Amy Metcalfe, Peter Faris, Hude Quan, Suzanne Tough, Deborah A. McNeil

**Affiliations:** a Department of Community Health Sciences, Cumming School of Medicine, University of Calgary, 3280 Hospital Drive NW, Calgary, AB T2N4Z6, Canada; b Alberta Health Services, 10101 Southport Road SW, Calgary, AB T2W3N2, Canada; c Department of Paediatrics, Cumming School of Medicine, University of Calgary, 3280 Hospital Drive NW, Calgary, AB T2N4Z6, Canada; d Department of Obstetrics and Gyneacology, Cumming School of Medicine, University of Calgary, 3280 Hospital Drive NW, Calgary, AB T2N4Z6, Canada; e Faculty of Nursing, University of Calgary, 2500 University Drive NW, Calgary, AB T2N1N4, Canada

**Keywords:** 1H2P, 1 hospitalization, 2 physician claims, ANOVA, analysis of variance, AOF, All Our Families, CI, confidence interval, EPDS, Edinburgh Postnatal Depression Scale, IQR, interquartile range, OR, odds ratio, PPD, postpartum depression, SD, standard deviation, Perinatal depression, Screening, Public health, Evaluation

## Abstract

Affecting 10–15% of women, postpartum depression (PPD) can be debilitating and costly. While early identification has the potential to improve timely care, recommendations regarding the implementation of routine screening are inconsistent. In Alberta, screening is completed using the Edinburgh Postnatal Depression Scale during public health well child clinic visits. The objective of this study was to examine the effectiveness of screening in identifying, diagnosing and treating women at increased risk for PPD over the first year postpartum, compared to those unscreened. The All Our Families prospective pregnancy cohort was linked to public health, inpatient, outpatient, physician claims and community pharmaceutical data over the first year postpartum. Descriptive statistics and bivariate analyses examined differences in sample characteristics and PPD and non-PPD related utilization by screening category. Odds ratios and 95% confidence intervals for PPD diagnosis and mental health drugs dispensed were generated using crude and multivariable logistic regression models. Within our sample, 87% of the eligible population were screened, with 3% receiving a high-risk score, and 13% were unscreened. Compared to those unscreened, women screened high-risk had higher odds of being diagnosed with PPD (OR: 3.88, 95% CI: 2.18–6.92) and women screened low/moderate-risk had reduced odds of receiving a diagnosis (OR: 0.51, 95% CI: 0.35–0.74). High-risk women had an increased likelihood of diagnosis, higher PPD-related utilization and drugs dispensed compared to those unscreened. This information suggests that screening was effective at streamlining resources in Alberta. Future work should focus on evaluating the cost-effectiveness of PPD screening.

## Highlights

• Postpartum depression affects 10-15% of women, representing the most significant complication following childbirth.
• Current Canadian guidelines do not recommend routine screening for perinatal depression in primary care settings.
• In Alberta, women are screened for PPD during public health well child clinic visits.
• If screened high-risk for PPD, women are offered referral to their family physician for follow-up diagnosis and treatment.
• This study suggests that screening was effective at streamlining resources in Alberta.
• High-risk women were more likely to be diagnosed with PPD compared to those unscreened.

## 1 Introduction

Postpartum depression (PPD) is characterized as major depressive disorder with an onset of symptoms occurring between delivery and one year postpartum (Hoertel et al., 2015). With a prevalence of 10–15% (O'Hara and Swain, 1996), PPD is costly and debilitating, affecting women's quality of life, social functioning and productivity (Myers et al., 2013). Left untreated, PPD increases the risk of negative consequences for the mother, child, and family (Davey et al., 2011; Lanes et al., 2011; Kingston et al., 2014). PPD is also associated with increased health service utilization (Fleury et al., 2014; Myers et al., 2013). According to the 2009 Institute of Medicine Report, greater service utilization tracking is required for mothers with PPD and children to better understand the relationship between required and unrequired resource use (Myers et al., 2013; Committee on Depression, 2009).

Screening has the potential to identify symptoms related to undiagnosed PPD that would otherwise remain untreated or be treated at a later, more severe, stage (Myers et al., 2013; Iragorri and Spackman, 2018). Early identification through routine screening has the ability to improve timely PPD care and optimize family well-being (Myers et al., 2013; Beck, 2001) while potentially reducing costs at a health system level (Iragorri and Spackman, 2018). However, globally, inconsistencies exist in routine screening recommendations, including the use of specific screening instruments and cut-off scores (Myers et al., 2013). Further, evidence is poor regarding rates of PPD diagnosis and treatment subsequent to screening (van der Zee-van Den Berg et al., 2017; Yawn et al., 2012b; Thombs et al., 2014). The Canadian Task Force for Preventive Health Care does not recommend routine screening for perinatal depression in primary care settings (Joffres et al., 2013), whereas the National Institute for Health and Care Excellence encourages the use of two-question screening where indicated (The National Institute for Health and Care Excellence, 2018), and clinical practice recommendations in the United States encourage universal screening when appropriate supports are in place (Committee on Obsetric Practice, 2015; Earls, 2010).

### 1.1 Screening pathway for PPD in Alberta, Canada

In Alberta, Canada, public health nurses use the Edinburgh Postnatal Depression Scale (EPDS) to opportunistically screen women during their first regular well child clinic visit, unless there is an indication to screen earlier (Alberta Health Services, 2019). The EPDS is a 10-item self-administered tool that takes <15 min to complete and provides public health nurses with a quantifiable and interpretable score (Cox et al., 1987; Liberto, 2012; Sword et al., 2008). As the most widely used instrument for PPD screening, the EPDS is available in several languages (Myers et al., 2013; Matthey et al., 2006; Mauri et al., 2010; O'Connor et al., 2016; Sword et al., 2008). Using a cut-off of 12/13, sensitivity is reported to range from 0.67 to 1.00 and specificity at 0.87 or more (O'Connor et al., 2016). At the time of this study, a score of 0–10 (of 30) on the EPDS indicated low-risk, 11 indicated moderate-risk and a score of 12 or greater indicated high-risk for PPD. Public health nurses used this criteria as a guide for their conversations, where women were informed about available resources and services, and, if high-risk, offered referral to her family physician for further diagnosis and treatment (Alberta Health Services, 2019). Of note, women within the moderate-risk range for PPD may or may not be referred to her family physician for care, as this decision is left at the discretion of the public health nurse (Alberta Health Services, 2019).

The objective of the current study was to examine the effectiveness of screening in identifying, diagnosing and treating women at increased risk for PPD over the first year postpartum, compared to those unscreened. Linked administrative data was used to determine PPD and non-PPD related health utilization patterns and to examine the association between PPD screening and 1) diagnosis and 2) mental health drugs dispensed up to 12 months postpartum, while controlling for sociodemographic characteristics and other risk factors for PPD. We considered screening *effective* if women screened high-risk were more likely to receive a PPD diagnosis, had higher PPD-related utilization and mental health drugs dispensed. Although effectiveness has been operationalized this way before (van der Zee-van Den Berg et al., 2017), to our knowledge this is the first study examining PPD screening effectiveness in this manner using linked administrative data in a Canadian perinatal sample.

## 2 Methods

Ethics approval was received from the Conjoint Health Research Ethics Board, University of Calgary (Ethics ID 140427).

The All Our Families (AOF; n = 3387) study was established in 2008 as a community-based pregnancy cohort grounded in Calgary, Alberta, and designed to examine early life influences on child development and family well-being (McDonald et al., 2013). Recruitment, eligibility, and data collection for the cohort have been previously reported (McDonald et al., 2013; Tough et al., 2017). Briefly: expectant women were recruited for the cohort between 2008 and 2011 prior to reaching 25 weeks' gestation, using a multi-method recruitment strategy involving community posters, primary health centres and city-wide public health laboratory services (McDonald et al., 2013). Two questionnaires were completed during pregnancy, two during the first postpartum year (at 4 and 12 months postpartum), and when children were two, three, and five years of age; data collection is ongoing for the eight year questionnaire (Tough et al., 2017).

### 2.1 Data linkage and sample

**Fig. 1:**
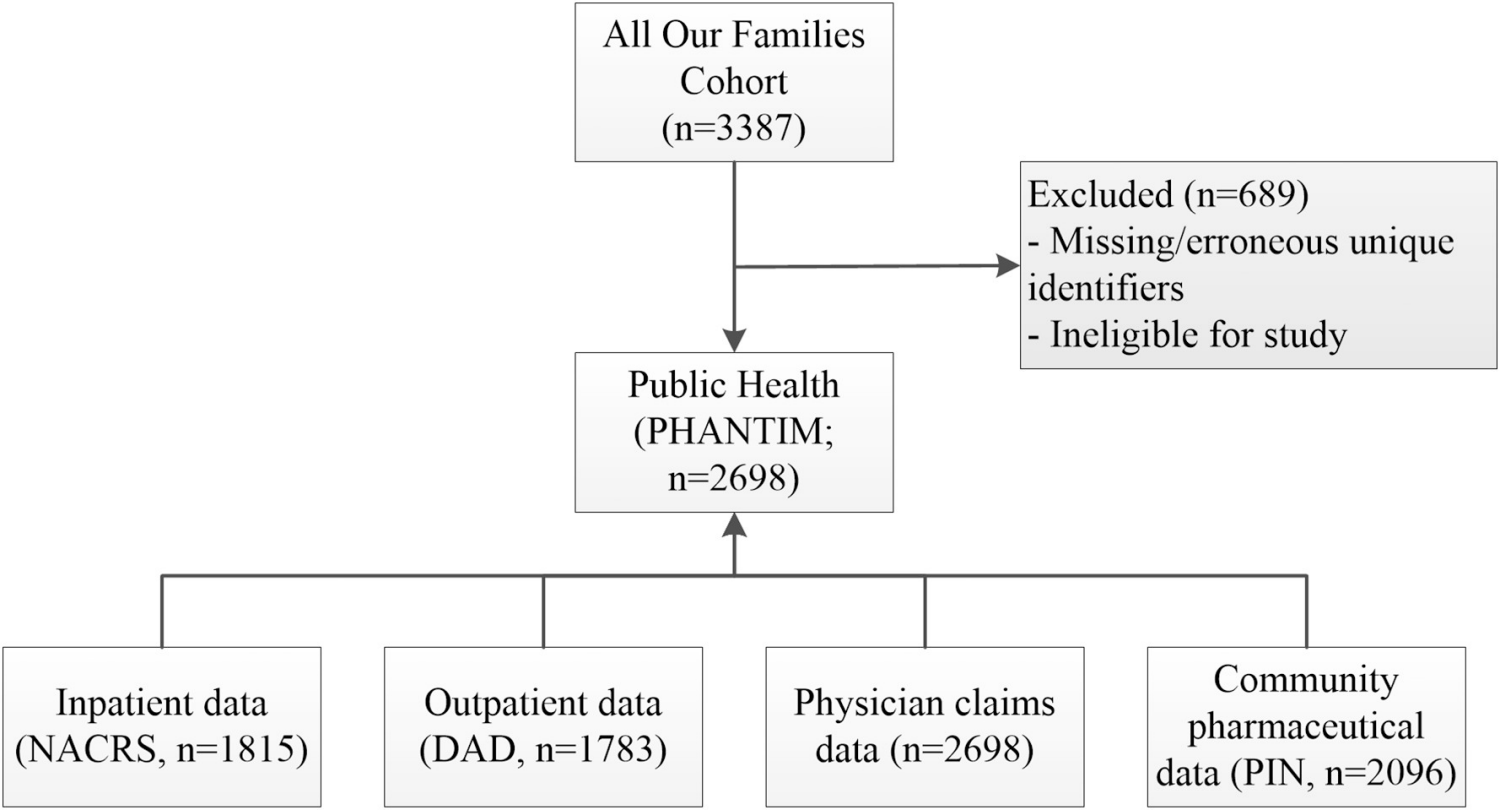
Administrative data linkage.

Women's self-reported demographic and other characteristics were obtained from AOF and linked using unique identifiers to public health data that contained information on EPDS screening and referral status. Data were further linked to physician claims, inpatient, outpatient, and community pharmaceutical databases to support identification of PPD diagnoses, PPD and non-PPD related utilization, and pharmacological treatment patterns (Fig. 1). Diagnosis and treatment status were examined up to 12 months postpartum subsequent to being offered screening; utilization was examined for the entire first year postpartum. Given that screening is an approach designed to capture women not yet diagnosed with PPD (Thombs et al., 2014), women were eligible for this study if they 1) were not receiving community pharmaceutical treatment for depression at the time of screening and 2) had no history of depression in the current pregnancy prior to screening; both were determined by examining PPD diagnostic information and mental health drugs dispensed to women between the date of delivery and date of screening. After removal of women with erroneous or missing unique identifiers and application of the eligibility criteria, the final sample was 2698.

### 2.2 Research objective and questions

The objective of the current study was to examine the effectiveness of screening in identifying, diagnosing and treating women at increased risk for PPD over the first year postpartum. Specific research questions included:

1. What are the patterns of PPD and non-PPD healthcare utilization for each screening category over the first year postpartum?
2. Is there an association between screening category and PPD diagnosis over the first year postpartum?
3. Is there an association between screening category and mental health drugs dispensed over the first year postpartum?

### 2.3 Exposure and outcome variables

Our primary exposure variable was screening status at two months postpartum. Using public health data, women's EPDS scores were obtained and they were categorized as being low/moderate-risk for PPD (scores 0–11), high-risk for PPD (scores ≥12), or unscreened. Outcome variables included PPD diagnosis and mental health drugs dispensed over the first year postpartum.

Psychiatrists and perinatal mental health experts identified and finalized the mental health codes included in the case definition for PPD (Appendix 1). Similar to Fiest et al. (2014), the team incorporated a broad range of mental health ICD codes comprising perinatal distress. These codes included six diagnostic categories: episodic mood disorder, anxiety disorder, stress reaction, adjustment reaction, depressive disorder, and other mood disorders. PPD *diagnosis* was defined using the 1 hospitalization, 2 physician claims (1H2P) method over a one year period, validated for hypertension and adopted by Health Canada in their Canadian Chronic Disease Surveillance System (Quan et al., 2009; Blais and Rochette, 2015). PPD *treatment* was defined according to whether a mental health drug recommended through Canadian treatment guidelines was dispensed to women within the first year postpartum (Kennedy et al., 2009; Canadian Psychiatric Association, 2006; Katzman et al., 2014). PPD and non-PPD related *utilization* were defined as the number of physician, inpatient, and outpatient visits, and unique drugs dispensed between birth and 12 months postpartum. Of note: all PPD-related utilization took place following a woman's screening date (if they were screened) or following delivery (if they were unscreened), in accordance with the eligibility criteria.

### 2.4 Statistical analysis

Descriptive statistics were produced using means and standard deviations (SD), medians and interquartile ranges (IQR), or frequencies, proportions and 95% confidence intervals (CI). Chi-square, Kruskal-Wallis non-parametric and analysis of variance (ANOVA) parametric tests examined differences in sample characteristics and PPD and non-PPD related utilization by screening category. Odds ratios (ORs) and 95% CI for PPD diagnosis and mental health drugs dispensed were generated using crude and multivariable logistic regression models. For the diagnosis and mental health drugs dispensed models, the unscreened category constituted the reference group in line with the Canadian guideline systematic literature review criteria, which supports quasi-experimental studies with an unscreened group (Joffres et al., 2013). Covariates entered in the initial models included maternal age, income, parity, ethnicity, self-reported maternal history of depression and comorbidities. We examined comorbidities as these increase the likelihood of healthcare utilization (Charlson et al., 2007, Charlson et al., 2014). We used the enhanced version of the Charlson comorbidity index in physician claims data and the Quan version for inpatient and outpatient data to identify the presence of medical comorbidities, such as diabetes, cancer, or cardiovascular disease, over the first year postpartum (Charlson et al., 1987). A binary variable was generated using the information retrieved from the index to categorize women as having or not having medical comorbidities. Statistical significance was set at p < 0.05. All variables of interest were included in the multivariable analysis and a manual backwards stepwise approach was used to arrive at final, parsimonious regression models. Data linkage, cleaning and analysis took place using Stata 15 (Stata Corp. 2015. Stata Statistical Software: Release 15. College Station, TX, USA: Stata Corp LP).

## 3 Results

**Fig. 2:**
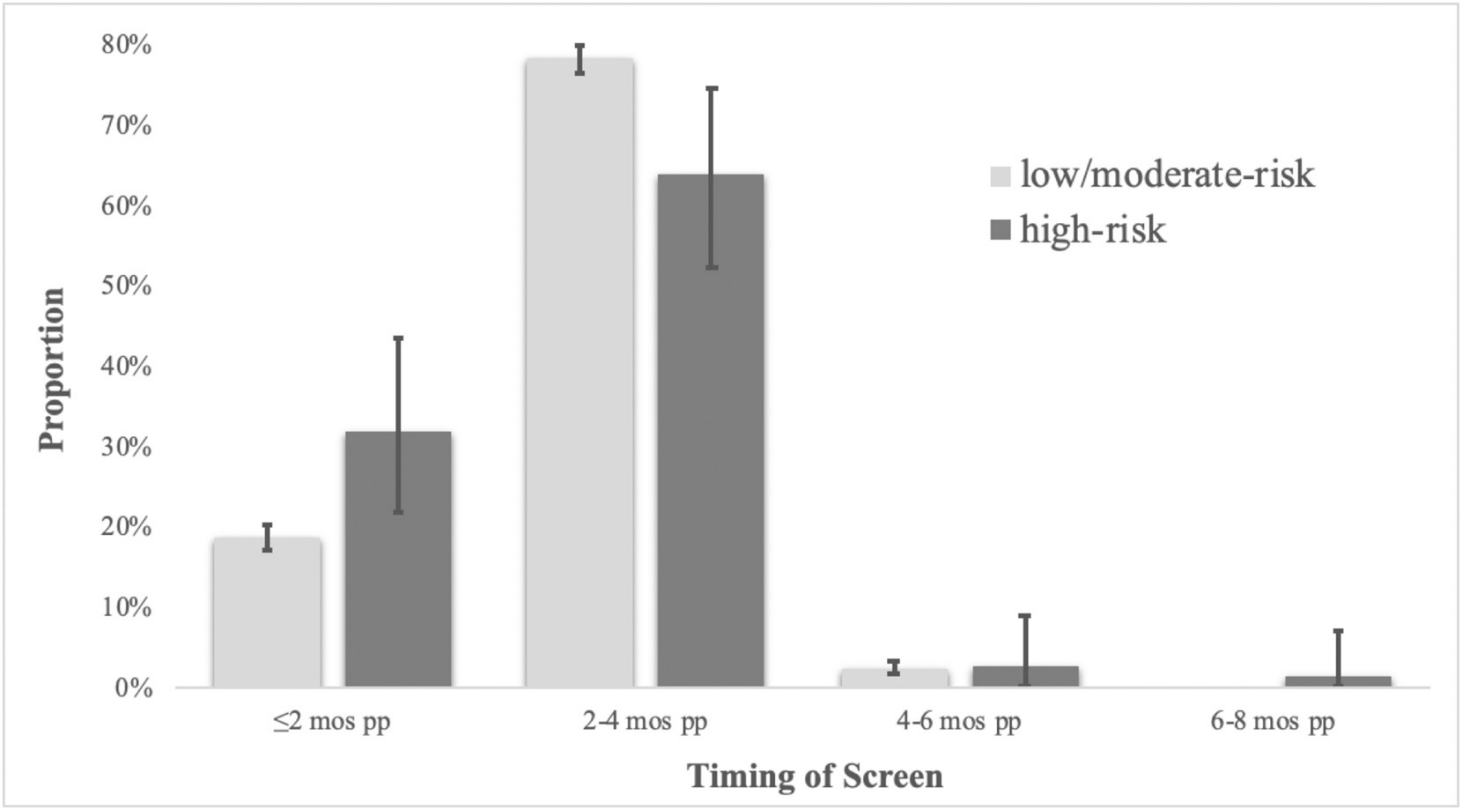
Timing of PPD screen and proportion scored low/moderate-risk or high-risk, with 95% CI.

Women were, on average, 31 years old and 89% had completed some college, university or graduate school (Table 1). Ninety-five percent were married or common-law, and two-thirds had a household income of $80,000 or more. The majority of participants were born, or had lived, in Canada >5 years (89%), while 78% were Caucasian. Of the women in our sample, 2261 (84%) received a low/moderate-risk score for PPD and 78 (3%) received a high-risk score. Thirteen percent of women were unscreened. Of those screened as high-risk, two-thirds were screened between two and four months postpartum and one-third were screened prior to two months postpartum (Fig. 2). Among those screened as low/moderate-risk for PPD, three-quarters were screened between two and four months postpartum, while 19% were screened prior to two months postpartum. Those screened as high-risk visited their physician for a PPD-related reason at a median of 21 days post screen (IQR: 56), while those screened low/moderate-risk visited their physician for a PPD-related reason at a median of 112 days post screen (IQR: 132) and unscreened women visited their physician for a PPD-related reason at a median of 50 days post-delivery (IQR: 129; p < 0.0001).

**Table 1:** Sample characteristics.

	Full sample (n = 2698)	Low/moderate-risk (n = 2261)	High-risk (n = 78)	Unscreened (n = 359)	p-Value
	Mean (SD)				
Maternal age at delivery	31.25 (4.40)	31.16 (4.35)	30.89 (4.69)	31.89 (4.59)	**0.015**
Education	n (%, 95% CI)				
High school or less	275 (10.2, 9.08–11.4)	236 (10.4, 9.2–11.8)	11 (14.1, 7.3–23.8)	28 (7.8, 5.2–11.1)	0.139
Some or complete university/college	1975 (73.2, 71.5–74.9)	1648 (72.9, 71.0–74.7)	57 (73.1, 61.8–82.5)	270 (75.2, 70.4–79.6)	
Some or complete graduate school	412 (15.3, 13.9–16.7)	346 (15.3, 13.8–16.9)	6 (7.7, 2.9–16.0)	60 (16.7, 13.0–21.0)	
Marital status					
Married/common law	2559 (94.8, 93.9–95.7)	2148 (95.0, 94.0–95.9)	74 (94.9, 87.4–98.6)	337 (93.9, 90.7–96.1)	0.482
Other	126 (4.7, 3.9–5.5)	101 (4.5, 3.7–5.4)	4 (5.1, 1.4–12.6)	21 (5.8, 3.7–8.8)	
Income (annual household)					
<$40,000	212 (7.9, 6.9–8.9)	157 (6.9, 5.9–8.1)	13 (16.7, 9.2–26.8)	42 (11.7, 8.6–15.5)	**<0.001**
$40,000–$79,999	548 (20.3, 18.8–21.9)	448 (19.8, 18.2–21.5)	15 (19.2, 11.2–29.7)	85 (23.7, 19.4–28.4)	
≥$80,000	1821 (67.5, 65.7–69.3)	1557 (68.9, 66.9–70.8)	44 (56.4, 44.7–67.6)	220 (61.3, 56.0–66.3)	
Time in Canada					
Born in Canada/lived here ≥5 years	2390 (88.6, 87.3–89.8)	2013 (89.0, 87.7–90.3)	63 (80.8, 70.3–88.8)	314 (87.5, 83.6–90.7)	0.067
Lived in Canada <5 years	261 (9.7, 8.6–10.9)	207 (9.2, 8.0–10.4)	12 (15.4, 8.2–25.3)	42 (11.7, 8.6–15.5)	
Parity					
Primaparous	1316 (48.8, 46.9–50.7)	1133 (50.1, 48.0–52.2)	37 (47.4, 36.0–59.1)	146 (40.7, 35.5–45.9)	**0.002**
Multiparous	1330 (49.3, 47.4–51.2)	1083 (47.9, 45.8–50.0)	38 (48.7, 37.2–60.3)	209 (58.2, 52.9–63.4)	
Ethnicity					
White/Caucasian	2096 (77.7, 76.1–79.2)	1761 (77.9, 76.1–79.6)	49 (62.8, 51.1–73.5)	286 (79.7, 75.1–83.7)	**0.014**
Other	566 (21.0, 19.4–22.6)	469 (20.7, 19.1–22.5)	26 (33.3, 23.1–44.9)	71 (19.8, 15.8–24.3)

Within our sample, high-risk women were slightly younger compared to those identified as low/moderate-risk or unscreened (p = 0.015). No differences by screening category existed in terms of education, marital status or time in Canada. However, women with lower income were more likely to score high-risk for PPD or be unscreened (p < 0.001), multiparous women were more likely to be unscreened (p = 0.002) and non-Caucasian women were more likely to receive a high-risk PPD score (p = 0.014).

### 3.1 Utilization patterns

Table 2 presents PPD and non-PPD related utilization among women over the first year postpartum. Overall, PPD-related utilization was associated with screening category for all types of health services; 51% of women screened high-risk visited their physician at least once for a PPD-related reason, compared to 16% of women screened low/moderate-risk and 17% of those unscreened (p < 0.001). Similarly, 9% of those screened high-risk had at least one PPD-related outpatient visit, compared to <2% of those screened low/moderate-risk and unscreened (p < 0.001), and 18% of women screened high-risk received a mental health drug compared to 5% of women screened low/moderate-risk and 9.5% of those unscreened (p < 0.001).

**Table 2:** PPD and non-PPD related utilization among women in the first year postpartum.

	Low/moderate-risk (n = 2261) n (%, 95% CI)	High-risk (n = 78) n (%, 95% CI)	Unscreened (n = 359) n (%, 95% CI)	p-Value
Utilization for PPD-related reason				
Physician visits				
0 claims	1893 (83.7, 82.1–85.2)	38 (48.7, 37.2–60.3)	297 (82.7, 78.4–86.5)	**<0.001**
1 claim	228 (10.1, 8.9–11.4)	14 (18.0, 10.2–28.3)	20 (5.6, 3.4–8.5)	
2 claims	61 (2.7, 2.1–3.5)	6 (7.7, 2.9–16.0)	12 (3.3, 1.7–5.8)	
3+ claims	79 (3.5, 2.8–4.3)	20 (25.6, 16.4–36.8)	30 (8.4, 5.7–11.7)	
Outpatient visits				
0 visits	2248 (99.4, 99.0–99.7)	71 (91.0, 82.4–96.3)	352 (98.1, 96.0–99.2)	**<0.001**
1 visit	5 (0.2, 0.1–0.5)	1 (1.3, 0–6.9)	1 (0.3, 0–1.5)	
2 visits	1 (0, 0–0.2)	2 (2.6, 0.3–9.0)	2 (0.6, 0–2.0)	
3+ visits	7 (0.3, 0.1–0.6)	4 (5.1, 1.4–12.6)	4 (1.1, 0.3–2.8)	
Inpatient visits^a^				
0 visits	2261 (100, 99.8–100)	77 (98.7, 93.1–100)	357 (99.4, 98.0–99.9)	**<0.001**
1 visit	0	1 (1.3, 0–6.9)	2 (0.6, 0.1–2.0)	
2 visits	0	0	0	
3+ visits	0	0	0	
Mental health drugs dispensed (unique prescriptions only)				
0 drugs	2144 (94.8, 93.8–95.7)	64 (82.0, 71.7–89.8)	325 (90.5, 87.0–93.4)	**<0.001**
1 drug	72 (3.2, 2.5–4.0)	9 (11.5, 5.4–20.8)	21 (5.8, 3.7–8.8)	
2 drugs	27 (1.2, 0.8–1.7)	2 (2.6, 0.3–9.0)	6 (1.7, 0.6–3.6)	
3+ drugs	18 (0.8, 0.5–1.3)	3 (3.8, 0.8–10.8)	7 (1.9, 0.8–4.0)	

Utilization for non-PPD related reason				
Physician claims				
0 claims	0	0	0	**0.003**
1 claim	8 (0.4, 0.1–0.7)	0	7 (1.9, 0.8–4.0)	
2 claims	8 (0.4, 0.1–0.7)	0	0	
3+ claims	2245 (99.2, 98.9–99.6)	78 (100, 95.4–100)	352 (98.1, 96.4–99.4)	
Outpatient visits				
0 visits	753 (33.3, 31.4–35.3)	26 (33.3, 23.1–44.9)	109 (25.4, 25.6–35.4)	0.062
1 visit	850 (37.6, 35.6–39.6)	26 (33.3, 23.1–44.9)	128 (35.6, 30.7–40.9)	
2 visits	325 (14.4, 13.0–15.9)	6 (7.7, 2.9–16.0)	60 (16.7, 13.0–21.0)	
3+ visits	333 (14.7, 13.3–16.3)	20 (25.6, 16.4–36.8)	62 (17.2, 13.5–21.6)	
Inpatients visits^a^				
0 visits	2182 (96.5, 95.7–97.2)	76 (97.4, 91.0–99.7)	341 (95.0, 92.2–97.0)	0.240
1 visit	37 (1.6, 1.2–2.2)	2 (2.6, 0.3–9.0)	6 (1.7, 0.6–3.6)	
2 visits	0	0	0	
3+ visits	42 (1.9, 1.3–2.5)	0	12 (3.3, 1.7–5.8)	
Drugs dispensed (unique prescriptions only)				
0 drugs	2154 (95.3, 94.3–96.1)	66 (84.6, 74.7–91.8)	326 (90.8, 87.3–93.6)	**<0.001**
1 drug	9 (0.4, 0.2–0.8)	0	3 (0.8, 0.2–2.4)	
2 drugs	16 (0.7, 0.4–1.1)	4 (5.1, 1.4–12.6)	3 (0.8, 0.2–2.4)	
3+ drugs	82 (3.6, 2.9–4.5)	8 (10.3, 4.5–19.2)	27 (7.5, 5.0–10.8)	

a Excludes visits for a labour and delivery reason (ICD10: Z37).

Screening category was significantly associated with non-PPD related utilization for physician visits and non-mental health drugs dispensed. Fifteen percent of women who screened high-risk, compared to 5% of those who screened low/moderate-risk and 9% of those unscreened received a non-mental health related drug over the first year postpartum (p < 0.001).

### 3.2 Multivariable regression analysis results

Overall, 8% of the sample was diagnosed with PPD, including 37% of women screened high-risk, 6% screened low/moderate-risk, and 12% of women that were unscreened (p < 0.001). Compared to those unscreened, women screened high-risk were more likely to receive a PPD diagnosis over the first year postpartum (OR: 3.88, 95% CI: 2.18–6.92; Table 3), whereas those screened low/moderate-risk had a 49% reduction in their likelihood of being diagnosed with PPD (OR: 0.51, 95% CI: 0.35–0.74). Further, women screened low/moderate-risk, compared to those unscreened, were half as likely to receive a mental health drug over the first year postpartum (OR: 0.53, 95% CI: 0. 36–0.80), whereas those screened high-risk were equally likely to receive a mental health drug as those unscreened (OR: 1.73, 95% CI: 0.86–3.46).

**Table 3:** Multivariable regression model results.

	Crude OR (95% CI)	Adjusted OR (95% CI)^a^
Outcome: PPD diagnosis		
Screening status		
Screened high-risk	**4.47 (2.56–7.83)**	**3.88 (2.18–6.92)**
Screened low-risk	**0.50 (0.35–0.72)**	**0.51 (0.35–0.74)**
Unscreened	1.00	1.00
Lifetime history of depression		
Yes	–	**3.01 (2.25–4.03)**
No	–	1.00
Presence of comorbidities^b^		
Yes	–	**1.78 (1.15–2.75)**
No	–	1.00

Outcome: mental health drugs dispensed		
Screening status		
Screened high-risk	1.98 (0.99–3.97)	1.73 (0.86–3.46)
Screened low-risk	**0.51 (0.34–0.76)**	**0.53 (0.36–0.80)**
Unscreened	1.00	1.00
Lifetime history of depression		
Yes	–	**2.96 (2.15–4.09)**
No	–	1.00
Presence of comorbidities^b^		
Yes	–	**1.88 (1.18–3.00)**
No	–	1.00

a Variables of interest entered in the initial models included maternal age, income, parity, ethnicity, lifetime history of depression and comorbidity.

b Presence of comorbidities defined using the Charlson comorbidity index applied to physician claims, inpatient and outpatient data.

## 4 Discussion

In our study, screening appeared to effectively separate the high-risk from low/moderate-risk women and to streamline resources between these groups; women screened low/moderate-risk had lower PPD and non-PPD related utilization, on average, compared to those screened high-risk. These results align with previous research on this topic, which found that women with depression symptomology were high utilizers of family physician services in the first two months postpartum (Dennis, 2004). Women screened high-risk also followed-up with their physician for a PPD-related reason much more quickly post-screen and were more likely to be diagnosed with PPD compared to those unscreened, which also aligns with other studies of a similar nature (Yawn et al., 2012a; Leung et al., 2010; van der Zee-van Den Berg et al., 2017).

Of those unscreened, 17% of women saw a physician at least once for a PPD-related visit and, compared to those identified as high-risk, were equally likely to receive a mental health drug over the first year postpartum, indicating they may have experienced symptoms of PPD despite being unscreened. This suggests there could be a missed opportunity for those who are unscreened whereby they may require additional opportunities for care or alternative screening, such as easy access clinics or a brief screen rather than a 10-item scale (Hewitt et al., 2009). Although reasons for being unscreened were inconsistently recorded in our data, further research examining reasons for not screening would be valuable to better understand utilization patterns among this group.

High-risk women appeared to have greater non-PPD related physician claims and drug utilization compared to those within the low/moderate-risk range and those that were unscreened, although it is unlikely that the differences in non-PPD related physician claims are clinically significant despite being statistically significant. Women with a high-risk score also had an indication of higher non-PPD related outpatient utilization, supporting the need to better understand whether their overall utilization was appropriate and necessary, as identified in the literature (Myers et al., 2013; Committee on Depression, 2009; Dennis, 2004; Roberts et al., 2001; Petrou et al., 2002). The Charlson comorbidity index does not identify pregnancy and postpartum conditions, which made it difficult to determine whether women were appropriately receiving care for postpartum complications (Charlson et al., 1987). Additional research to better understand these patterns over a longer period of time is warranted. Results from our multivariable analysis further confirmed that marital status, socioeconomic status, and parity were not generally associated with PPD once other factors were taken into consideration, whereas history of depression was moderately associated with PPD, as identified through previous research (Myers et al., 2013; Beck, 2001), and women with comorbidities also had an increased risk for a PPD diagnosis compared to women without. This also aligns with findings from the literature (Mitra et al., 2016; Katon et al., 2007).

According to our findings, of those screened high-risk, only 51% had a visit with a physician for a PPD-related reason. While screening itself may increase awareness and potentially prompt some women to seek help from sources other than her physician, for example through peer support, internet-based self-help or phone support (Kingston et al., 2014), low physician visit rates among those screened high-risk may be alleviated through increased communication between public health and primary care around PPD risk scores, decreasing women's risk of not receiving appropriate follow-up care subsequent to her high-risk screening score. This is further supported within the literature, where women have noted their frustration with disconnected care pathways and have indicated they would prefer to be contacted and supported by their family physicians following a public health screen (Sword et al., 2008; Kingston et al., 2014).

### 4.1 Strengths and limitations

Strengths of this study included the use of linked survey and administrative data, which enabled us to conduct a pragmatic evaluation of screening effectiveness (Virnig and McBean, 2001). Application of our exclusion criteria also allowed for isolation of the screening exposure, a design recommendation for examination of efficacy (Thombs et al., 2014; Joffres et al., 2013). Despite these strengths, there are limitations to this study. Over 600 women from AOF were not linked to the administrative data. Analysis showed that those not linked had lower levels of education and income and were less likely to be partnered, raising the potential for selection bias. However, this likely underestimates the effects found in our study. Of note, ‘low education and income’ in our study identifies women who are under the median annual income of $80,000; it could be that not having a safety net or making ends meet in an affluent urban climate creates a context of added stress for these families. Further supports such as postpartum peer support and community engagement are worthy of exploration given their importance in previous work with this cohort (McDonald et al., 2016a; McDonald et al., 2016b). Another limitation is the potential for non-differential misclassification of exposure and outcome status due to coding and/or data entry errors; this would also lead to an underestimate of effects found in this study. Although the literature supports using the Charlson comorbidity index to control for confounding in epidemiological data, it has traditionally been used to predict mortality risk, healthcare utilization and costs (Schneeweiss et al., 2001). The validity of using this index to adjust for the presence versus absence of comorbidities is a potential direction for future research. Additionally, only 3% of those in our sample were screened high-risk for PPD. This is a great deal lower than the prevalence of PPD in the general population, reported to be between 10 and 15% (O'Hara and Swain, 1996), and suggests a much lower sensitivity for the EPDS than is reported elsewhere (O'Connor et al., 2016). Future research should focus on assessing the adequacy of the cut-off score in Canadian women. Lastly, although the literature suggests women prefer non-pharmaceutical treatment for PPD (Dennis and Chung-lee, 2006), we were unable to evaluate whether women received this type of treatment in our study. Future work examining PPD screening and non-pharmaceutical treatment patterns among Albertan women may be warranted.

## 5 Conclusion

The present study provides encouraging evidence for the effectiveness of PPD screening in Alberta; women screened high-risk were more likely to receive a PPD diagnosis and had higher PPD-related utilization and drugs dispensed compared to those unscreened. Future work should focus on evaluating the cost-effectiveness of screening across the entire treatment pathway.

The following is the supplementary data related to this article.

Appendix 1 PPD case definition. Appendix 1

## Ethics approval and consent to participate

Ethics approval was received from the Conjoint Health Research Ethics Board, University of Calgary (Ethics ID 140427).

## Conflicts of interest

The authors declare they have no conflicts of interest.

## Authors' contributions

SP contributed to the conception, methodology, analysis, interpretation, visualization, drafting the original manuscript, and review and editing of the manuscript. SWM contributed to the conception, methodology, funding acquisition, project administration, data acquisition, interpretation, visualization, supervision and review and editing of the manuscript. AM, PF, and HQ contributed to the methodology, interpretation, visualization and review and editing of the manuscript. ST contributed to the funding acquisition, data curation, interpretation, review and editing of the manuscript. DAM contributed to the conception, methodology, funding acquisition, data acquisition, interpretation, visualization, supervision and review and editing of the manuscript.

## References

[bb0005] Alberta Health Services (2019). Postpartum depression. https://www.albertahealthservices.ca/info/Page16138.aspx.

[bb0010] Beck C.T. (2001). Predictors of postpartum depression: an update. Nurs. Res..

[bb0015] Blais C., Rochette L. (2015). Trends in prevalence, incidence and mortality of diagnosed and silent coronary heart disease in Quebec. Health Promot. Chronic Dis. Prev. Can..

[bb0020] Canadian Psychiatric Association (2006). Clinical practice guidelines. Management of anxiety disorders. Can. J. Psychiatr..

[bb0025] Charlson Mary E., Pompei Peter, Ales Kathy L., MacKenzie Ronald (1987). A new method of classifying prognostic comorbidity in longitudinal studies: development and validation. J. Chronic Dis..

[bb0030] Charlson Mary, Charlson Robert E., Briggs William, Hollenberg James (2007). Can disease management target patients most likely to generate high costs? The impact of comorbidity. J. Gen. Intern. Med..

[bb0035] Charlson Mary, Wells Martin T., Ullman Ralph, King Fionnuala, Shmukler Celia (2014). The Charlson comorbidity index can be used prospectively to identify patients who will incur high future costs. PLoS One.

[bb0040] Committee on Depression, Parenting Practices and the Healthy Development of Children. 2009. “Depression in Parents, Parenting, and Children: Opportunities to Improve Identification, Treatment, and Prevention.” Edited by MJ England and Leslie J Sim. Washington, DC: The National Academies Press. doi:10.17226/12565. 25009931

[bb0045] Committee on Obstetric Practice (2015). The American College of Obstetricians and Gynecologists Committee Opinion no. 630. Screening for perinatal depression. Obstet. Gynecol..

[bb0050] Cox J.L., Holden J.M., Sagovsky R. (1987). Detection of postnatal depression. Br. J. Psychiatry.

[bb0055] Davey Heather L., Tough Suzanne C., Adair Carol E., Benzies Karen M. (2011). Risk factors for sub-clinical and major postpartum depression among a community cohort of Canadian women. Matern. Child Health J..

[bb0060] Dennis C.-L. (2004). Influence of depressive symptomatology on maternal health service utilization and general health. Arch Womens Ment Health.

[bb0065] Dennis Cindy-lee, Chung-lee Leinic (2006). Postpartum depression help-seeking barriers and maternal treatment preferences: a qualitative systematic review. Birth.

[bb0070] Earls M.F. (2010). Incorporating recognition and management of perinatal and postpartum depression into pediatric practice. Pediatrics.

[bb0075] Fiest Kirsten M., Jette Nathalie, Quan Hude, St Germaine-Smith Christine, Metcalfe Amy, Patten Scott B., Beck Cynthia A. (2014). Systematic review and assessment of validated case definitions for depression in administrative data. BMC Psychiatry.

[bb0080] Fleury Marie-Josée, Grenier Guy, Bamvita Jean-Marie, Caron Jean (2014). Determinants and patterns of service utilization and recourse to professionals for mental health reasons. BMC Health Serv. Res..

[bb0085] Hewitt C., Gilbody S., Brealey S., Paulden M., Palmer S., Mann R., Green J. (2009). Methods to identify postnatal depression in primary care: an integrated evidence synthesis and value of information analysis. Health Technol. Assess..

[bb0090] Hoertel Nicolas, Lopez Saioa, Peyre Hugo, Wall Melanie M., Gonzalez-Pinto Ana, Limosin Frederic, Blanco Carlos (2015). Are symptom features of depression during pregnancy, the postpartum period and outside the peripartum period distinct? Results from a nationally representative sample using item response theory (IRT). Depress Anxiety.

[bb0095] Iragorri Nicolas, Spackman Eldon (2018). Assessing the value of screening tools: reviewing the challenges and opportunities of cost-effectiveness analysis. Public Health Rev..

[bb0100] Joffres Michel, Jaramillo Alejandra, Dickinson James, Lewin Gabriela, Pottie Kevin, Shaw Elizabeth, Gorber Sarah Connor, Tonelli Marcello (2013). Recommendations on screening for depression in adults. CMAJ.

[bb0105] Katon W., Lin E.H., Kroenke K. (2007). The association of depression and anxiety with medical symptom burden in patients with chronic medical illness. Gen. Hosp. Psychiatry.

[bb0110] Katzman Martin A., Bleau Pierre, Blier Pierre, Chokka Pratap, Kjernisted Kevin, Ameringen Michael Van, Antony Martin M. (2014). Canadian clinical practice guidelines for the management of anxiety, posttraumatic stress and obsessive-compulsive disorders. BMC Psychiatry.

[bb0115] Kennedy Sidney H., Lam Raymond W., Parikh Sagar V., Patten Scott B., Ravindran Arun V. (2009). Canadian Network for Mood and Anxiety Treatments (CANMAT) clinical guidelines for the management of major depressive disorder in adults. J. Affect. Disord..

[bb0120] Kingston Dawn, McDonald Sheila, Tough Suzanne, Austin Marie-Paule, Hegadoren Kathy, Lasiuk Gerri (2014). Public views of acceptability of perinatal mental health screening and treatment preference: a population based survey. BMC Pregnancy Childbirth.

[bb0125] Lanes Andrea, Kuk Jennifer L., Tamim Hala (2011). Prevalence and characteristics of postpartum depression symptomatology among Canadian women: a cross-sectional study. BMC Public Health.

[bb0130] Leung Shirley S.L., Leung Cynthia, Lam T.H., Hung S.F., Chan Ruth, Yeung Timothy, Miao May (2010). Outcome of a postnatal depression screening programme using the Edinburgh Postnatal Depression Scale: a randomized controlled trial. J Public Health (Oxf).

[bb0135] Liberto Terri L. (2012). Screening for depression and help-seeking in postpartum women during well-baby pediatric visits: an integrated review. J. Pediatr. Health Care.

[bb0140] Matthey S., Henshaw C., Elliott S., Barnett B. (2006). Variability in use of cut-off scores and formats on the Edinburgh Postnatal Depression Scale - implications for clinical and research practice. Arch Womens Ment Health.

[bb0145] Mauri Mauro, Oppo Annalisa, Montagnani Maria Sole, Borri Chiara, Banti Susanna, Camilleri Valeria, Cortopassi Sonia, Ramacciotti Daniele, Rambelli Cristina, Cassano Giovanni B. (2010). “Beyond “postpartum depressions”: specific anxiety diagnoses during pregnancy predict different outcomes: results from PND-ReScU.”. J Affect Disord.

[bb0150] McDonald Sheila W., Lyon Andrew W., Benzies Karen M., McNeil Deborah a, Lye Stephen J., Dolan Siobhan M., Pennell Craig E., Bocking Alan D., Tough Suzanne C. (2013). The all our babies pregnancy cohort: design, methods, and participant characteristics. BMC Pregnancy Childbirth.

[bb0155] McDonald Sheila W., Kehler Heather, Bayrampour Hamideh, Fraser-Lee Nonie, Tough Suzanne (2016). Risk and protective factors in early child development: results from the All Our Babies (AOB) pregnancy cohort. Res. Dev. Disabil..

[bb0160] McDonald Sheila W., Kehler Heather L., Tough Suzanne C. (2016). Protective factors for child development at age 2 in the presence of poor maternal mental health: results from the All Our Babies (AOB) pregnancy cohort. BMJ Open.

[bb0165] Mitra M., Iezzoni L.I., Zhang J., Long-Bellil L.M., Smeltzer S.C., Barton B.A. (2016). Prevalence and risk factors for postpartum depression symptoms among women with disabilities. Matern. Child Health J..

[bb0170] Myers Evan R., Aubuchon-Endsley Nicki, Bastian Lori A., Gierisch Jennifer M., Kemper Alex R., Swamy Geeta K., Wald Marla F. (2013). Efficacy and Safety of Screening for Postpartum Depression.

[bb0175] O'Connor Elizabeth, Rossom Rebecca C., Henninger Michelle, Groom Holly C., Burda Brittany U. (2016). Primary care screening for and treatment of depression in pregnant and postpartum women evidence report and systematic review for the US preventive services task force. JAMA.

[bb0180] O'Hara Michael W., Swain Annette M. (1996). Rates and risk of postpartum depression–a meta-analysis. Int Rev Psychiatry.

[bb0185] Petrou S., Cooper P., Murray L., Davidson L. (2002). Economic costs of postnatal depression in a high risk British cohort. Br. J. Psychiatry.

[bb0190] Quan Hude, Khan Nadia, Hemmelgarn Brenda R., Tu Karen, Chen Guanmin, Campbell Norm, Hill Michael D., Ghali William A., McAlister Finlay A. (2009). Validation of a case definition to define hypertension using administrative data. Hypertension.

[bb0195] Roberts J., Sword W., Watt S., Gafni A., Krueger P., Sheehan D., Soon-Lee K. (2001). Costs of postpartum care: examining associations from the Ontario mother and infant survey. Can J Nurs Res.

[bb0200] Schneeweiss Sebastian, Seeger John D., Maclure Malcolm, Wang Philip S., Avorn Jerry, Glynn Robert J. (2001). Performance of comorbidity scores to control for confounding in epidemiologic studies using claims data. Am. J. Epidemiol..

[bb0205] Sword Wendy, Busser Dianne, Ganann Rebecca, McMillan Theresa, Swinton Marilyn (2008). Women's care-seeking experiences after referral for postpartum depression. Qual. Health Res..

[bb0210] The National Institute for Health and Care Excellence (2018). Depression in adults: recognition and management (CG90). https://www.nice.org.uk/guidance/cg90.

[bb0215] Thombs Brett D., Arthurs Erin, Coronado-Montoya Stephanie, Roseman Michelle, Delisle Vanessa C., Leavens Allison, Levis Brooke (2014). Depression screening and patient outcomes in pregnancy or postpartum: a systematic review. J. Psychosom. Res..

[bb0220] Tough Suzanne C., McDonald Sheila W., Collisson Beverly Anne, Graham Susan A., Kehler Heather, Kingston Dawn, Benzies Karen (2017). Cohort profile: the All Our Babies pregnancy cohort (AOB). Int. J. Epidemiol..

[bb0225] Virnig Beth A., McBean Marshall (2001). Administrative data for public health surveillance and planning. Annu. Rev. Public Health.

[bb0230] Yawn B.P., Dietrich A.J., Wollan P., Bertram S., Graham D., Huff J., Kurland M. (2012). TRIPPD: a practice-based network effectiveness study of postpartum depression screening and management. Ann. Fam. Med..

[bb0235] Yawn Barbara P., Olson Ardis L., Bertram Susan, Pace Wilson, Wollan Peter, Dietrich Allen J. (2012). Postpartum depression: screening, diagnosis, and management programs 2000 through 2010. Depress. Res. Treat..

[bb0240] van der Zee-van Den Berg Angarath I., Boere-Boonekamp Magda M., IJzerman Maarten J., Haasnoot-Smallegange Riet M.E., Reijneveld Sijmen A. (2017). Screening for postpartum depression in well-baby care settings: a systematic review. Matern. Child Health J..

