# Supplementary material for: Examining postpartum depression screening effectiveness in well child clinics in Alberta, Canada: A study using the All Our Families cohort and administrative data
Source: Prev Med Rep. 2019 May 3;14:100888. doi: 10.1016/j.pmedr.2019.100888 (PMC6517566; doi:10.1016/j.pmedr.2019.100888)
Supplement: Appendix 1 — PPD case definition. [file mmc1.pdf]

**Appendix 1:** PPD Case Definition	
Diagnostic Category	**ICD Codes**
Episodic Mood Disorder	ICD 9 Codes: 296, 296.2, 296.5, 296.6, 296.9<br>ICD 10 Codes: F31.8 F31.9
Anxiety Disorder	ICD 9 Codes: 300, 300.1, 300.3, 300.4, 300.9<br>ICD 10 Codes: F40.0, F41.0, F41.1, F41.2, F41.9, F60.5
Stress Reaction	ICD 9 Codes: 308, 308.3, 308.9<br>ICD 10 Codes: F43.9
Adjustment Reaction	ICD 9 Codes: 309, 309.1, 309.2, 309.9<br>ICD 10 Codes: F43.2
Depressive Disorder	ICD 9 Codes: 311, 296.3<br>ICD 10 Codes: F32.1, F32.2, F32.9, F33.1, F33.8, F33.9
Other Mood Disorder	ICD 9 Codes: 648.4, 298.9<br>ICD 10 Codes: F34.0, F39, F53.0, R45.8, O99301
